# Caregiver Perspectives on Pain Sensitivity and Pain Experience in Rett Syndrome

**DOI:** 10.1080/24740527.2023.2229400

**Published:** 2023-07-28

**Authors:** B. J. Byiers, A. M. Merbler, A. Raiter, C. C. Burkitt, F. J. Symons

**Affiliations:** a Department of Educational Psychology, University of Minnesota, Minneapolis, Minnesota, USA; b Gillette Children’s, St. Paul, Minnesota, USA

**Keywords:** Rett syndrome, caregiver report, intellectual and developmental disabilities, pain burden, chronic pain

## Abstract

**Background:**

Although delayed or decreased responses to pain are commonly reported among caregivers of individuals with Rett syndrome (RTT), previous studies in relatively small samples have documented that caregivers are concerned about pain, particularly due to gastrointestinal and musculoskeletal conditions.

**Aims:**

The purpose of the current study was to investigate in detail caregivers’ perceptions of pain sensitivity, as well as the types, severity, and effect of pain experienced by individuals with RTT in a larger sample than previous studies.

**Methods:**

A total of 51 caregivers (mostly mothers) participated in the study, which involved standardized questionnaires and interviews. The individuals with RTT ranged in age from 2 to 52 years of age, and most (*n* = 46; 90%) met criteria for classic RTT.

**Results:**

Across the sample, 84% of caregivers reported that they believed that their child was less sensitive to pain compared to her typically developing peers. Despite this perception, 63% of caregivers reported that their child had experienced at least one form of pain in the previous 7 days, and 57% reported their child experienced at least one form of chronic pain. On average, caregivers reported that their child’s pain was of moderate severity and interfered with at least one activity of daily living.

**Conclusions:**

The results suggest that pain is a substantial concern among caregivers of individuals with RTT and indicate that additional research is needed to understand the apparent paradox of frequently reported pain experiences despite widespread perceptions of decreased pain sensitivity.

## RÉSUMÉ

**Contexte:** Bien que des réponses tardives ou diminuées à la douleur soient fréquemment signalées par les soignants de personnes atteintes du syndrome de Rett (RTT), des études antérieures dans des échantillons relativement petits ont documenté que les soignants étaient préoccupés par la douleur, en particulier en raison de troubles gastro-intestinaux et d’affections musculosquelettiques.

**Objectifs:** Le but de la présente étude était d’étudier en détail les perceptions de la sensibilité à la douleur par les soignants ainsi que les types, la gravité et l’effet de la douleur ressentie par les personnes atteintes de RTT dans un échantillon plus important que les études précédentes.

**Méthodes:** Au total, 51 soignants (principalement des mères) ont participé à l’étude, qui a eu recours à des questionnaires et à des entretiens standardisés. Les personnes atteintes de RTT étaient âgées de 2 à 52 ans, et la plupart (n = 46 ; 90 %) répondaient aux critères de la RTT classique.

**Résultats:** Dans l’échantillon, 84 % des soignants ont déclaré qu’ils croyaient que leur enfant était moins sensible à la douleur par rapport à ses pairs qui se développent normalement. Malgré cette perception, 63 % des soignants ont signalé que leur enfant avait ressenti au moins une forme de douleur au cours des sept jours précédents, et 57 % ont déclaré que leur enfant avait souffert d’au moins une forme de douleur chronique. En moyenne, les soignants ont signalé que la douleur de leur enfant était d’intensité modérée et interférait avec au moins une activité de la vie quotidienne.

**Conclusions:** Les résultats indiquent que la douleur est une préoccupation importante chez les soignants des personnes atteintes de RTT et que des études supplémentaires sont nécessaires pour comprendre le paradoxe apparent voulant que des expériences douloureuses soient fréquemment rapportées bien que la perception d’une diminution de la sensibilité à la douleur soit largement répandue.

Rett syndrome (RTT) is an X-linked neurodevelopmental condition primarily caused by loss-of-function mutations of the X-linked methyl-CpG-binding protein 2 gene (MECP2).^1^ The estimated global prevalence of RTT is approximately five to ten cases per 100,000 females (males are rarely affected).^2^ The classic phenotype of RTT is characterized by seemingly normal pre- and perinatal development until approximately 6 to 18 months of age, at which point affected individuals experience a severe developmental regression. This regression leads to loss of speech, loss of purposeful hand use, development of gait abnormalities and hand stereotypies, and intellectual disability.^3^ The clinical spectrum of RTT associated with MECP2 mutations also includes milder presentations in which hand function and language are somewhat preserved, as well as more severe presentations that may be apparent from birth, particularly among males.^4^

Several comorbid conditions are common in RTT, including gastrointestinal issues, scoliosis, seizures, breathing disturbances, sleep disorders, and dental problems.^5–10^ Some of these conditions (e.g., gastrointestinal dysfunction, seizures) occur relatively early in development, whereas others (e.g., scoliosis and other musculoskeletal complications) frequently worsen over time. Nevertheless, survival into the fifth decade of life is typical for individuals with RTT with appropriate medical care.^11^ With these health problems and increasing survival, there is reason to believe that pain may be a frequent occurrence for many individuals with RTT, particularly at older ages.

Due to the severe motor and communication impairments associated with RTT, pain may be difficult to recognize and treat in this population. As in most populations with communication challenges, caregivers, typically parents, are generally relied upon to identify when their child is experiencing pain, decide when pain is of sufficient concern to seek treatment, and assist in evaluating the effectiveness of pain treatment strategies.^12^ As with any self- or proxy report measure, however, assessment of pain by caregivers is inescapably colored by the caregiver’s perception of and opinions about the individual, as well as their own experiences with pain and treatments. A further challenge to the identification and treatment of pain in RTT and related syndromes is the perception that affected individuals have diminished sensitivity to pain. Decreased or delayed reactions to pain have been documented anecdotally in literature,^13,14^ and “diminished response to pain” is currently a supportive criterion for diagnoses of atypical RTT.^3^ In a study comparing parent-reported pain behaviors among individuals with RTT, cerebral palsy, and autism spectrum disorders and typically developing controls,^15^ the individuals with RTT were reported to show the fewest behavioral pain indicators across all of the groups. In a separate large-scale survey of parents of females with confirmed MECP2 mutations, 65% reported that their child showed decreased reactions to pain.^16^ Interestingly, the authors of the study noted that this perceived change in pain sensitivity appeared to be somewhat specific to “external” pain, such as pain from accidental injury or injections. In contrast, some parents noted possible increased sensitivity to “internal” pain due to issues such as constipation or headaches. The authors also noted that decreased pain sensitivity was least likely to be reported by parents of girls in the youngest age group.

Consistent with the idea that individuals with RTT may be sensitive to internal sources of pain, Symons et al.^17^ reported that 24% of caregivers who had a child with RTT reported that their child had experienced pain in the previous 30 days. In another sample, Barney et al.^18^ reported that 89% (*n* = 16) of girls and women living with RTT had experienced at least one type of pain in the previous week. Both studies were limited by their small sample sizes, and neither examined parents’ perceptions of pain sensitivity.

Ultimately, the literature surrounding caregiver perceptions of pain sensitivity, sources, and burden in individuals with RTT is limited and not entirely clear but suggests that the burden of pain may be high despite the apparently altered or ambiguous pain responses. Caregivers are relied upon as proxy reporters, and there is likely a complex interaction among their perceptions about the condition of RTT itself, their own child, and their child’s lifetime of experiences with different noxious events or chronic health conditions. Given the reliance on caregivers, the goal of this study is to utilize a relatively large sample of caregiver proxy reports to further investigate their perceptions of pain sensitivity in RTT and the types, severity, and impact of pain experienced.

## Materials and Methods

### Participants and Recruitment

Appropriate ethics approval was obtained for this study from the University of Minnesota (Institutional Review Board No. 006640), and informed consent was obtained from the parent or legal guardian of each participant. The consent process was conducted either in person or over the phone, with digital signatures on virtual forms. Participant dyads consisting of primary caregivers and their child/ward with RTT were recruited from an independent specialty children’s rehabilitation hospital or through posting of recruitment materials on social media pages of national RTT parent advocacy organizations. Dyads were eligible to participate if the primary caregiver spoke English with adequate proficiency to complete the questionnaires and resided within the United States and the affected individual had a confirmed pathogenic loss-of-function mutation of MECP2 and/or a clinical diagnosis of classic RTT without previous genetic testing.

### Procedures

After informed consent was obtained, questionnaires were completed via a semistructured interview (Dalhousie Pain Interview [DPI] only) and standardized survey questions. Clinical evaluations were conducted by researchers with experience in RTT based on in-person or remote video conferencing observations of the individuals.

#### Measures

##### Demographic Questionnaire

Caregivers were asked to report on the age, gender, race, genetic and clinical diagnosis, and gross motor ability of the individual with RTT (Table 1).

**Table 1.** Clinical and demographic characteristics of the participants with RTT and their caregivers.

Characteristic		12 and younger					13 and older						
		Mild			Severe		Mild			Severe		Total	
		Median	Min-max		Median	Min-max	Median	Min-max		Median	Min-max	Median	Min-max
Total *N*		12			8		9			22		51	
Age		6.3	2.3–10.0		6.6	2.8–10.8	18.1	13.1–52.5		21.5	13.8–39.2	14.3	2.3–52.5
Clinical severity score		11	5–15		19	16–27	12	5–15		22	16–31	18	5–31
		*N*	%		*N*	%	*N*	%		*N*	%	*N*	%
Biological mother as reporter		12	100		7	88	8	89		16	73	43	84
Ambulation													
	With assistance	3	25		1	13	1	11		4	18	9	18
	Without assistance	5	42		3	38	8	89		3	14	19	37
Seizures													
	Previous/controlled	6	50		3	38	1	11		6	27	16	31
	Uncontrolled	1	8		3	38	2	22		13	59	19	37
Genetic mutation													
	p.Arg133Cys	1	8		0	0	0	0		3	14	4	8
	p.Pro152Arg	1	8		0	0	0	0		1	5	2	4
	p.Thr158Met	2	17		1	13	0	0		0	0	3	6
	p.Arg306Cys	0	0		0	0	1	11		0	0	1	2
	Other missense	3	25		1	13	2	22		0	0	6	12
	p.Arg168*	0	0		1	13	0	0		0	0	1	2
	p.Arg270*	2	17		2	25	1	11		1	5	6	12
	p.Arg294*	1	8		0	0	0	0		4	18	5	10
	Other truncating	1	8		0	0	2	22		3	14	6	12
	Deletion/insertion	1	8		2	25	1	11		5	23	9	18
	Not reported*	0	0		1	13	1	11		4	18	6	12
	Not tested	0	0		0	0	1	11		1	5	2	4

##### Pain Sensitivity

To characterize pain sensitivity as perceived by caregivers, respondents were asked to rate the participant’s pain sensitivity relative to peers without disabilities in three categories: more sensitive, less sensitive, or the same level of sensitivity.

##### Dalhousie Pain Interview

The DPI^19^ was used to assess the type and general description, potential cause, duration, and intensity of pain participants had experienced in the previous 7 days. The survey consists of ten items that were repeated for each type of pain reported and was collected via a semistructured interview. Caregivers were asked to identify the source of the participant’s pain, which was then categorized by the researcher as accidental; gastrointestinal; musculoskeletal; neurological (i.e., headaches or seizure-related pain); procedural, equipment, or therapy related, which may include any pain resulting from previous surgery or treatment (e.g., pain associated with hardware from spinal fusion); illness or infection; other; or unknown. Pain intensity for each type of pain was rated on an 11-point numeric rating scale (0 = *no pain*, 10 = *worst pain possible*). For descriptive purposes, caregivers’ pain ratings were categorized into mild (ratings of 1 to 4), moderate (ratings of 5 to 7), or severe (ratings of 8 to 10) based on previous studies.^20,21^ Numeric pain ratings for recent pain experiences were not provided by two caregivers.

Using the DPI, chronic pain experience was also documented. Chronic pain was defined as pain that may come or go but that first occurred at least 6 months previously. Caregivers were asked to describe the participant’s pain source and identify the onset of their chronic pain. Caregivers were also asked to describe the individual’s behavior on good pain and bad pain days and to rate the intensity of the pain on a bad day. Finally, caregivers were asked to report whether they believed the participant ever experienced pain-free days.

##### Brief Pain Inventory

The Brief Pain Inventory (BPI)^22^ was used to measure the extent to which pain interfered with 12 different activities of daily living in the previous 7 days. Activities of daily living asked about include general activity, mood, mobility, schoolwork or other chores, relations with others, sleep, enjoyment of life, self-care, recreational activities, social activities, communication with others, and learning new information. Caregivers used an 11-point numeric rating scale (0 = *pain did not interfere with that activity*, 10 = *pain completely interfered*) to report pain interference with the above activities, for a possible range of 0 to 120. Two caregivers did not complete the BPI measure due to time constraints, resulting in a sample size of 49 for this measure.

##### Clinical Severity

The Kerr Rett syndrome clinical severity scale^23^ was used to characterize the presence of comorbid health conditions and overall clinical severity. The clinical severity scale was completed by two researchers with experience working with individuals with RTT based on a combination of parent report and observation. This scale was selected over other available questionnaires for RTT because it includes multiple clinical domains that are relevant for both typical and atypical RTT, allows for comparisons across individuals, and can be completed based on a combination of caregiver report and direct clinical observation. Because information regarding early development and head circumference was unavailable for many participants, these items were excluded. The modified scale included 17 items, each rated from 0 (*no symptoms or very mild symptoms*) to 2 (*severe symptoms*), resulting in a possible scale range of 0 to 34.

### Data Analysis

Pain reports were characterized by pain type, and descriptive statistics (i.e., *N*, %) were calculated for each. Due to nonnormal distributions, medians and interquartile ranges were calculated for total scores on the BPI and pain intensity scores. To evaluate whether patterns of pain reports varied systematically across age and symptom severity, participants were divided into two RTT clinical severity categories: mild cases were defined as individuals with total scores of 15 or lower, and severe cases were defined as individuals with scores of 16 or higher. This cut point was selected based on the bimodal nature of the distribution of scores, with 15 being the approximate split point between the two peaks. Participants were also grouped into two age categories (i.e., 12 years and under; 13 years and older), because smaller groupings did not change the overall pattern of results. Due to the small cell sizes within groups, only descriptive statistics are reported.

## Results

A sample of 51 females with RTT (aged 2 to 52 years old) and their primary caregivers participated in the study. The caregivers were predominantly White and non-Hispanic (*n* = 48, 94%), with the remaining caregivers identifying as Black (*n* = 1, 2%), Asian (*n* = 1, 2%), and other (*n* = 1, 2%). No information on caregiver age, education level, or socioeconomic status was collected. Nearly all (*n* = 43, 84%) of the caregivers were biological mothers to the individual with RTT. The other caregivers included biological fathers (*n* = 4, 8%), professional caregivers (i.e., personal care assistants, group home staff; *n* = 2, 4%), one adoptive mother (2%), and one adoptive father (2%). Among the individuals with RTT, 46 participants met all criteria for classic RTT (90%), 2 met criteria for the preserved speech variant (4%), and 3 presented with a developmental course consistent with atypical RTT at the time of evaluation but were too young to be categorized (10%).^3^ All but two individuals had clinical diagnoses of classic RTT confirmed with genetic testing. See Table 1 for additional demographic and clinical information.

Most of the participants with RTT (*n* = 44, 86%) were taking at least one over-the-counter or prescription medication regularly. The most common medication class used was anti-epileptics (*n* = 37, 73%), followed by laxatives (*n* = 28, 55%), medications for sleep (*n* = 15, 29%), medications to treat acid reflux/gastroesophageal reflux disease (*n* = 15, 29%), medications to treat allergies and asthma (*n* = 12, 24%), medications to regulate mood (*n* = 10, 20%), hormonal birth control for menstrual regulation (*n* = 7, 14%), baclofen for spasticity (*n* = 4, 8%), statins for cholesterol control (*n* = 2, 4%), and levothyroxine (*n* = 2, 4%). Additional medications each being taken by a single participant included levodopa for Parkinsonism, immunosuppressant medications for autoimmune disorders, and beta blockers for hypertension. Five caregivers reported regular use of pain medications for their child/ward (10%), with use of ibuprofen reported in two cases (4%), acetaminophen in two cases (4%), and opioid medications in one case (2%).

**Figure 1.**
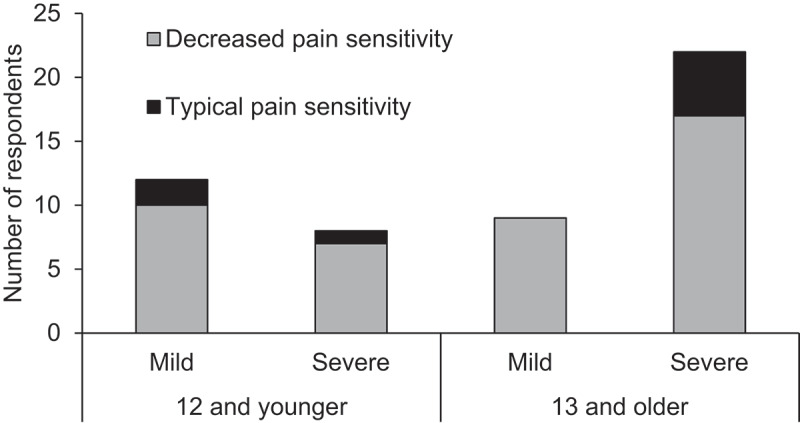
Frequency of reports of typical versus decreased pain sensitivity by age (12 and younger versus 13 and older) and clinical severity (mild versus severe).

Across the sample, 43 caregivers reported that their child/ward with RTT was less sensitive to pain than peers (84%). The remaining 8 reported they showed typical pain responses (16%). There were no clear characteristics that differentiated between these two groups: clinical severity scores for the “typical sensitivity” group ranged from 5 to 31 (out of a total of 34; median = 20.5; decreased sensitivity group median = 16.0; min = 5, max = 30), and ages ranged from 3.8 to 39.1 years (median = 14.3; decreased sensitivity group median = 14.3; min = 2.3, max = 52.5; see Fig. 1). Among the 20 individuals under the age of 12, 17 were reported to have decreased pain sensitivity (85%) compared to 26 of 31 of the individuals 13 and older (84%).

## Recent Pain

Despite the nearly ubiquitous perception of decreased pain sensitivity, pain experiences were frequently reported by caregivers. In total, 32 respondents reported at least one form of recent pain (63%; i.e., in the past 7 days), with 25 reporting a single form of pain (49%) and 7 reporting two forms of recent pain (14%). Gastrointestinal and musculoskeletal pain were the most reported. Gastrointestinal pain occurred at relatively consistent rates across the age and severity groups, whereas musculoskeletal pain was most prevalent in the older participants, particularly among those with more severe clinical symptoms. Neurological pain, therapy- or procedure-related pain, pain due to illness/infection, and other pain types were reported only among individuals in the severe group. In contrast, three of three reports of recent accidental pain and four of five reports of pain from unknown sources were among respondents in the mild group (see Table 2 for more details).

**Table 2.** Summary of reported chronic and recent pain sources by clinical severity group.

	12 and younger						13 and older						Total Sample					
	Mild		Severe		All		Mild		Severe		All		Mild		Severe		All	
Characteristic/pain type	*N*	%	*N*	%	*N*	%	*N*	%	*N*	%	*N*	%	*N*	%	*N*	%	*N*	%
Total *N*	12		8		20		9		22		31		21		30		51	
Decreased pain sensitivity	10	83	7	88	17	85	9	100	17	77	26	84	19	90	24	80	43	84
Recent pain (previous 7 days)																		
Any source	5	42	5	63	10	50	6	67	16	73	22	71	11	52	21	70	32	63
Gastrointestinal pain	2	17	2	25	4	20	0	0	3	14	3	10	2	10	5	17	7	14
Musculoskeletal	0	0	1	13	1	5	2	22	7	32	9	29	2	10	8	27	10	20
Neurological	0	0	1	13	1	5	0	0	3	14	3	10	0	0	4	13	4	8
Therapy or procedure related	0	0	0	0	0	0	0	0	4	18	4	13	0	0	4	13	4	8
Illness/infection	0	0	1	13	1	5	0	0	1	5	1	3	0	0	2	7	2	4
Accidental	2	17	0	0	2	10	1	11	0	0	1	3	3	14	0	0	3	6
Other source	0	0	0	0	0	0	2	22	2	9	4	13	2	10	2	7	4	8
Unknown source	2	17	1	13	3	15	1	11	0	0	1	3	3	14	1	3	4	8
Chronic pain																		
Any source	5	42	5	63	10	50	4	44	14	64	18	58	9	43	19	63	28	55
Gastrointestinal pain	3	25	3	38	6	30	0	0	7	32	7	23	3	14	10	33	13	25
Musculoskeletal	0	0	3	38	3	15	2	22	8	36	10	32	2	10	11	37	13	25
Neurological	0	0	1	13	1	5	0	0	3	14	3	10	0	0	4	13	4	8
Therapy or procedure related	0	0	0	0	0	0	0	0	3	14	3	10	0	0	3	10	3	6
Other source	0	0	0	0	0	0	1	11	5	23	6	19	1	5	5	17	6	12
Unknown source	2	17	1	13	3	15	2	22	0	0	2	6	4	19	1	3	5	10
Experiences pain at least daily	1	8	2	25	3	15	3	33	6	27	9	29	4	19	8	27	12	24

The median severity rating across all recent pain experiences reported was 5.0 (out of 10), consistent with moderate severity, with a range of 1 to 10. Among the 7 respondents in the mild clinical symptoms group, ratings ranged from 3 to 7 (median = 4.0), with 4 ratings in the mild range (57%) and 3 in the moderate range (43%). Among the 23 respondents in the severe clinical symptoms group, ratings ranged from 1 to 10 (median = 5.0), with 7 ratings in the mild range (30%), 13 in the moderate range (57%), and 3 in the severe range (13%). When the most severe recent pain was evaluated across both severity groups, 10 caregivers rated the pain as mild (20%; i.e., between 1 and 4), 31 rated the pain as moderate (28%; i.e., 5 to 7), and 3 rated the pain as severe (10%; i.e., between 8 and 10).

## Chronic Pain

**Figure 2.**
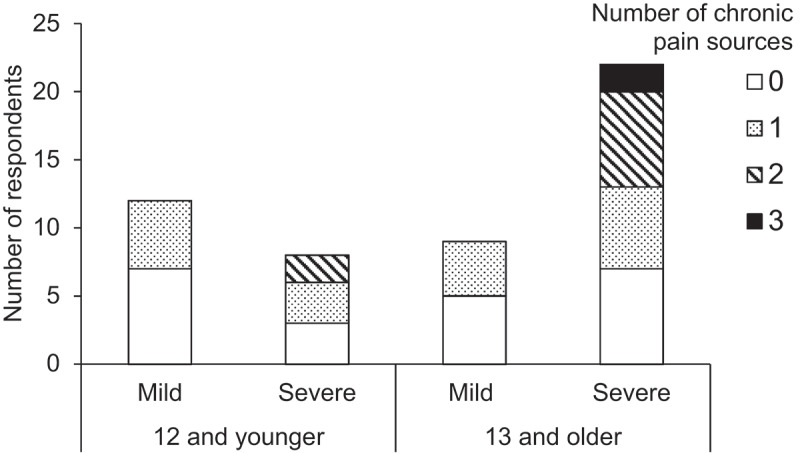
Number of reported chronic pain sources by age (12 and younger versus 13 and older) and clinical severity (mild versus severe).

Regarding chronic pain, 29 respondents reported at least one source (57%), 9 reported two sources (18%), and 2 reported three sources (4%), for a total of 42 reported sources. Musculoskeletal and gastrointestinal pain were the most common types of chronic pain, aligning with the recent pain experiences reported (see Table 2 for further details). Although chronic pain was relatively common across age and severity groups, no individuals in the mild clinical severity group were reported to have more than one type of chronic pain, whereas 11 respondents in the severe group reported two or three distinct forms of chronic pain (see Fig. 2). Twelve respondents reported that they believed the individual with RTT did not experience pain-free days, accounting for 41% of the participants with chronic pain, 29% of individuals 13 and older, and 24% of the full sample.

**Figure 3.**
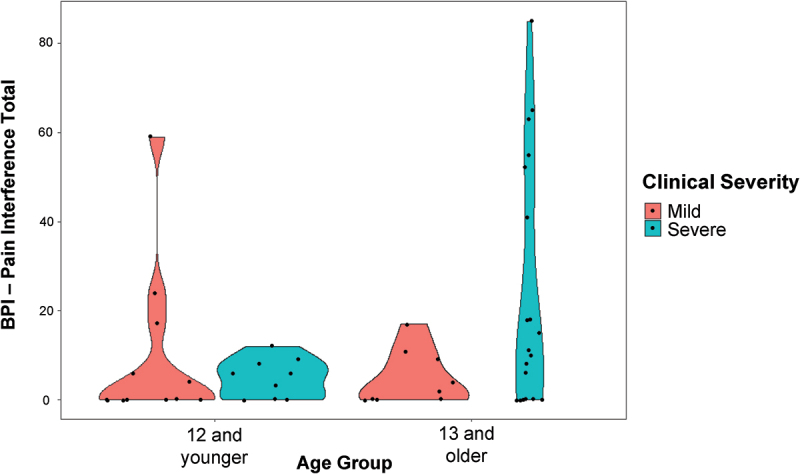
Violin plots representing the distribution of total pain interference scores on the BPI by age (12 and younger versus 13 and older) and clinical severity (mild versus severe). Points represent data from individual cases, and the shapes represent the density of the distribution across values of pain interference (i.e., wider = more values in that range).

All but one of the caregivers who reported that the participant had experienced pain in the past week reported that it had interfered with at least one activity of daily living on the BPI (*N* = 29, 97%). Within this group, the median total score for this sample was 11 and the mean was 21 (out of 120; min = 2, max = 85). Nine items were endorsed by at least half the sample reporting pain: mood (*N* = 27, 90%), enjoyment of life (*N* = 21, 70%), general activity (*N* = 20, 67%), mobility (*N* = 17, 57%), recreation (*N* = 17, 57%), social activities (*N* = 16, 53%), school, work or chores (*N* = 15, 50%), communication (*N* = 15, 50%), and sleep (*N* = 15, 50%). Three items were endorsed by less than half the sample: relations with others (*N* = 14, 47%), self-care (*N* = 14, 47%), and learning (*N* = 13, 43%). Apparent differences in pain interference were observed when the sample was grouped according to clinical severity (i.e., mild versus severe) and age (i.e., 12 years and under, 13 years and older). Specifically, high pain interference scores (i.e., 20 points and above) occurred only in the younger mild group and the older severe group, with the latter group showing the highest pain burden overall (see Fig. 3).

## Discussion

Caregivers of individuals with RTT reported nearly universally that they believed that their child/ward was less sensitive to pain compared to their peers, but they also frequently reported high occurrences of recent and chronic pain, particularly among individuals with more severe clinical presentations. Because few caregivers reported typical pain responses in their child/ward, it was not possible to evaluate in detail how these individuals might differ on the DPI/BPI from the larger. Additionally, 25% of caregivers reported that their child/ward never had pain-free days, indicating a high need for proper assessment and treatment to maintain and improve quality of life. There appears to be somewhat of a paradox in that individuals are being reported as less pain sensitive on the one hand but caregivers consistently report significant amounts of recent and chronic pain on the other hand. The issue, in part, for the field and for our scientific understanding of pain in RTT, is that our reliance on proxy report has proceeded (as in much of neurodevelopmental disorders clinical pain research) without detailed study of variables that may influence the proxy report. Knowing what influences the perception of “reduced sensitivity” while also reporting frequent pain experience for the same individual needs to be squared if we are to understand the variance in pain outcomes for RTT.

With respect to the most common sources of pain in RTT, the results of the current study are consistent with prior studies showing gastrointestinal and musculoskeletal pain to be relatively common.^9,17,18^ It is also worth noting that apparent pain from unknown sources was reported in approximately 10% of cases. Cases in which parents suspect pain but cannot identify a source require special consideration, because optimal pain management generally requires accurate identification of its source. Further, uncertainty regarding whether their child/ward is in pain may lead to increased stress for parents already at risk for chronic stress.^24^ It is also notable that interference with activities of daily living due to pain appears to increase with age, at least among individuals with more severe clinical presentations. This finding suggests a need for proactive strategies to treat emerging health conditions early to maintain quality of life.

All but one caregiver who reported that their child experienced pain reported that it interfered with at least one activity of daily living in this sample, indicating a high level of pain burden in daily life. Many of these activities are known factors for overall health and function, such as sleep and social interactions, and were impacted for more than half the sample reporting pain. This is consistent with the previous literature. A previous investigation of the impact of pain on quality of life for individuals with intellectual disabilities found that pain was associated with decreases in physical health, leisure activity, and negative behaviors.^25^ Similarly, Breau et al.^26^ reported that children with intellectual disability, including children with genetic syndromes, were able to perform more skills on pain-free compared to days they were in pain and that pain affected all areas of daily life surveyed (e.g., socialization, motor skills). Because a quarter of the present sample did not have pain-free days, it is crucial to better assess, treat, and, ultimately, prevent pain in RTT.

Several groups have reported on efforts to develop or adapt observational behavioral measurement systems to assess pain among individuals with RTT and other forms of intellectual and developmental disabilities.^27–30^ Development of such systems is of critical importance to advancing equitable pain treatment for these populations, and evidence suggests that these approaches can differentiate episodes of known pain from non-pain periods.^29^ Additional work is needed, however, to fully appreciate the degree to which behavioral signs reflect the actual pain experience of individuals with RTT. Although the current evidence suggests that individuals with RTT show fewer behavioral pain signs relative to other groups with and without neurodevelopmental disabilities, the source of this disparity remains unclear. There are at least two potential hypotheses: (1) reduction in MeCP2 results in decreased nociception and pain in this population, meaning that these individuals actually experience less pain, or (2) individuals with RTT experience pain in ways that are comparable to other groups but do not express the pain in the same ways owing to a combination of communication and motor deficits and decreased adaptive capacity. Both hypotheses have some theoretical and/or preliminary empirical support. MeCP2 is involved in several biological systems affecting nociception and analgesia (see Martin^31^ for a review). Altered tactile and pain sensitivity is a consistent finding across RTT animal models, ranging from zebra fish to primates,^32–36^ although the specific tactile modalities (i.e., mechanical versus thermal) and direction of effect (i.e., reduced versus increased sensitivity) vary across studies. This preclinical evidence suggests that nociception and tactile sensitivity may be affected in RTT, although the impact on pain sensitivity in humans remains hypothetical.

On the other hand, O’Leary et al.^37^ documented elevations in electrodermal activity and heart rate during venipuncture in a sample of five individuals with classic RTT, despite very small changes in behavioral reactivity. These results suggest that individuals with RTT show changes in physiological activity that are characteristic of pain responses despite not showing dramatic observable responses to nociceptive events. The authors speculated that this apparent lack of behavioral response might be attributable to motor impairment. In contrast, a study examining the behavioral responses of 17 individuals with RTT to a standardized set of calibrated tactile stimuli found that the overall level of behavioral reactivity was strongly predicted by the individual’s baseline heart rate variability. These results suggest that, in at least some cases, a lack of behavioral response to tactile experiences, including acute pain, may be due to a lack of autonomic capacity. This reduced capacity, in turn, results in an inability to mount an adaptive response to unexpected or novel events rather than a failure of perception/nociception in this population. Because both of these studies were small and preliminary, however, there is a need for further research investigating the potential mechanisms underlying the decreased behavioral reactivity to pain in RTT, as well as variability in behavioral and physiological responding across individuals. Finally, additional research is needed to elucidate the mechanisms behind the apparent differences in behavioral pain expression in response to internal and external pain sources.

In addition to pursuing research to elucidate the mechanisms underlying differences in pain experience and expression in this population, further research into how caregivers make judgments regarding others’ pain experience is urgently needed. There is an apparent disconnect between the interpretation of pain sensitivity and the reports of frequent, and often severe, pain events. Anecdotally, when asked about pain sensitivity and experience, caregivers frequently made comments such as “she doesn’t really show when she’s in pain, so when she lets me know, it must really hurt.” Such comments inherently recognize that pain expression may not be directly tied to pain experience and suggest that some caregivers believe that their child/ward has other painful experiences that may be missed. Caregivers also mentioned having been told by medical practitioners or other parents of children with RTT that girls and women with RTT do not feel pain, which leads them to question themselves when they perceive their child/ward as experiencing pain. Qualitative work may be beneficial in understanding the factors that shape parents’ perceptions of pain sensitivity, which in turn could lead to more informed approaches to pain assessment and treatment for the RTT population. There is substantial evidence from studies in the general population that cognitive, behavioral, and affective factors affect how people express pain and perceive pain in others,^38–41^ but few studies have investigated these factors in relation to caregiver reports of pain among individuals with neurodevelopmental disabilities.

Although this study has several strengths, including a relatively large and well-characterized sample, there are also several limitations. First, we were limited by an exclusive focus on caregiver proxy report. Though relying on proxy report is unavoidable in this sample, it is likely that there are biases operating on the part of the respondents. Secondly, this sample of participants was recruited from select sources and, as such, are not a random or representative sample of the population of individuals living with RTT or their caregivers. Future work should aim to include a more representative sample of individuals with RTT. Although based on the distributions of the data or general reasoning, comparisons by age and clinical severity were based on somewhat arbitrary cutoffs, and clinical severity scales such as the one used in this study may obscure interindividual differences by summing together distinct clinical features. Finally, although the sample size was relatively large for a rare disease population, the small cell sizes limited the degree to which subgroup analyses could be performed, particularly regarding the sample of caregivers who considered their child/ward to have typical pain sensitivity.

In summary, the results of the current study indicate that pain is a substantial concern among caregivers of individuals with RTT, despite widespread perceptions of decreased pain sensitivity in this population. Gastrointestinal and musculoskeletal pain are the most reported pain sources, and total pain burden appears to increase with age, at least among those with severe clinical presentations. Medical personnel working with individuals with RTT should therefore consider assessment of gastrointestinal and musculoskeletal issues presenting with possible pain and discomfort. A small but notable sample of respondents reported that their child/ward appeared to experience chronic pain for which they were unable to identify a source. These results raise concerns that individuals with RTT may be experiencing pain that goes unnoticed, and therefore untreated, likely due to a combination of caregiver perceptions and unusual or subtle pain behaviors. Medical providers should be aware that individuals with RTT may present with unusual or subtle pain behaviors, and changes in behavior should result in thorough medical evaluation for possible pain sources.

## Data Availability

The data that support the findings of this study are available on request from the corresponding author, BJB. The data are not publicly available because they contain information that could compromise the privacy of research participants.
